# Multimodal Contrastive Learning for Remote Sensing Image Feature Extraction Based on Relaxed Positive Samples

**DOI:** 10.3390/s24237719

**Published:** 2024-12-03

**Authors:** Zhenshi Zhang, Qiujun Li, Wenxuan Jing, Guangjun He, Lili Zhu, Shijuan Gao

**Affiliations:** 1 College of Basic Education, National University of Defense Technology, Changsha 410073, China; zhangzhenshi@nudt.edu.cn; 2 School of Geosciences and Info-Physics, Central South University, Changsha 410083, China; csu_lqq@csu.edu.cn (Q.L.); 215012162@csu.edu.cn (W.J.); 3 State Key Laboratory of Space-Ground Integrated Information Technology, Beijing Institute of Satellite Information Engineering, Beijing 100086, China; hgjun_2006@163.com; 4 Hunan Key Laboratory of Land Resources Evaluation and Utilization, Hunan Provincial Institute of Land and Resources Planning, Changsha 410083, China

**Keywords:** multimodal contrastive learning, identity constraint, positive sample relaxation

## Abstract

Traditional multimodal contrastive learning brings text and its corresponding image closer together as a positive pair, where the text typically consists of fixed sentence structures or specific descriptive statements, and the image features are generally global features (with some fine-grained work using local features). Similar to unimodal self-supervised contrastive learning, this approach can be seen as enforcing a strict identity constraint in a multimodal context. However, due to the inherent complexity of remote sensing images, which cannot be easily described in a single sentence, and the fact that remote sensing images contain rich ancillary information beyond just object features, this strict identity constraint may be insufficient. To fully leverage the characteristics of remote sensing images, we propose a multimodal contrastive learning method for remote sensing image feature extraction, based on positive sample tripartite relaxation, where the model is relaxed in three aspects. The first aspect of relaxation involves both the text and image inputs. By introducing learnable parameters in the language and image branches, instead of relying on fixed sentence structures and fixed image features, the network can achieve a more flexible description of remote sensing images in text and extract ancillary information from the image features, thereby relaxing the input constraints. Second relaxation is achieved through multimodal alignment of various features. By aligning semantic information with the corresponding semantic regions in the images, the method allows for the relaxation of local image features under semantic constraints. This approach addresses the issue of selecting image patches in unimodal settings, where there is no semantic constraint. The proposed method for remote sensing image feature extraction has been validated on four datasets. On the PatternNet dataset, it achieved a 91.1% accuracy with just one-shot.

## 1. Introduction

Traditional supervised learning methods [1,2,3,4] largely depend on manually labeled data, resulting in significant human and material resources for model training, often leading to lengthy training cycles. This is particularly unsuitable for high-resolution remote sensing imagery, which is characterized by rapid updates and large volumes of data. Consequently, self-supervised contrastive learning, which reduces reliance on manual labels and enables models to focus more on the details of the images, is gradually becoming a more appropriate paradigm for training high-resolution remote sensing interpretation models with vast and rapidly changing data.

Schroff and Yeh [5,6] proposed that the core of self-supervised learning lies in selecting an anchor sample, along with positive and negative samples related to the anchor. The model learns feature invariance and separability by minimizing the distance between the anchor and positive samples in feature space, while maximizing the distance between the anchor and negative samples. Zhao and Chao [7,8] took this further by generating positive samples through data augmentation of the anchor sample, considering any sample other than the anchor and its positive samples as a negative sample. And they thought that learning invariance means that features extracted from instances within the same category should remain consistent or similar, whereas learning separability implies that features extracted from different categories should show notable differences and be easily distinguishable. From this perspective, Radford et al. [9,10,11] later defined positive and negative samples as matched and unmatched “image-text” pairs when training multimodal models. By maximizing the similarity of matched samples in the feature space while minimizing the similarity between unmatched samples, the model can capture the feature invariance and separability inherent beyond multimodal data alignment.

However, the aforementioned definitions of contrastive learning are based on a strict identity assumption, where positive samples are strictly defined as augmented versions of the anchor sample itself, thus limiting feature extraction to the instance level [12,13]. This results in instances being pulled closer together in the feature space, while the distribution of different instances within the same class remains uncontrolled. Particularly in remote sensing imagery, significant variability among geographic entities of the same class renders the consideration of only the augmentations of the anchor sample as positive samples inadequate. To address this issue and better learn feature invariance, Khosla et al. [14,15,16,17,18] proposed a unimodal relaxation sample strategy, which involves constructing relaxed signals through clustering and prior knowledge to identify relaxed samples, thereby loosening the composition of positive samples. For multimodal contrastive learning, the relaxation strategy parallels that of unimodal learning, focusing on data augmentation for both images and texts [19,20,21,22]. The image augmentation methods align with those in unimodal contrastive learning, while text augmentation methods emphasize techniques such as reordering words and replacing synonyms.

However, the aforementioned multimodal relaxation strategy focuses on relaxation within a single modality, failing to create effective complementary relaxation between modalities and not fully utilizing the diverse information offered by multimodality. Moreover, due to the complexity and uncertainty of remote sensing imagery, manually designed sentences often struggle to accurately describe image content, and using global features of the images neglects their rich texture, spectral information, and other details [23,24].

To address these issues, we propose a three-way relaxation approach for multimodal contrastive learning in remote sensing, which involves relaxation across language, image, and image–language interaction. Specifically, regarding language, research by Zhou et al. [25] indicates that different sentences, or even different arrangements of words in the same sentence, can significantly impact the final outcome. This suggests that simple sentence enhancements, such as synonym replacement or reordering, are inadequate relaxation methods. Therefore, we design several learnable vectors at the language input level which relax the language input through continuous prompts, allowing the model to select the most suitable contextual prompts in the feature space. In terms of image processing, to capture the rich additional information inherent in high-resolution remote sensing imagery, we introduce learnable embedding vectors at each layer of the image input. Unlike traditional image augmentation, the content learned by these vectors can diverge from conventional image features, making them more suitable for the current network architecture. Finally, in the interaction between images and language, we treat image patches from different branches as positive samples to bring them closer together, thereby achieving relaxation of local image regions under semantic constraints.

## 2. Method

This section first outlines the overall framework of the multimodal contrastive learning method for remote sensing image feature extraction based on positive sample tripartite relaxation. It then provides a detailed explanation of two key modules in the framework: the relaxed identity input module in the bimodal setting and the semantic-guided relaxed positive sample selection module.

### 2.1. MRiSSNet

**Figure 1:**
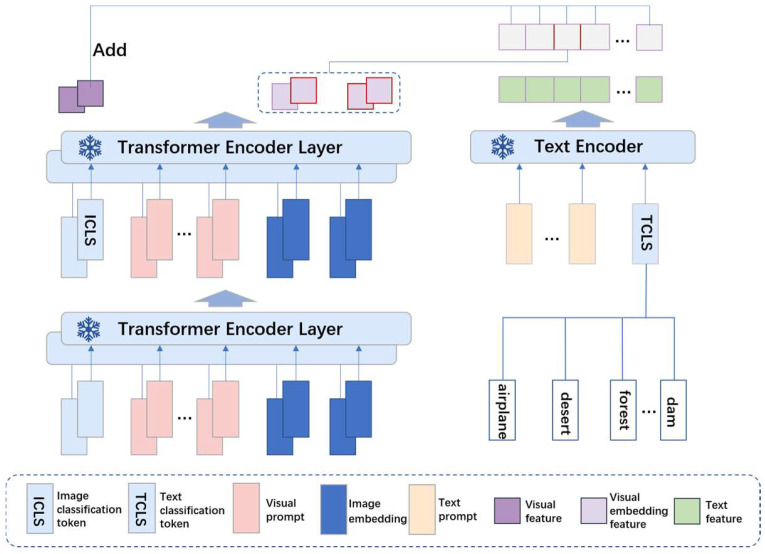
Overall structure diagram of MRiSSNet.

Based on multimodal contrastive learning under the relaxed identity assumption, we propose MRiSSNet (Multimodal Relaxed Identity Sample Strategy Network). This network consists of two image feature extraction networks and one text feature extraction network. By introducing prompts into both the image and language branches for relaxation and enabling interaction between the two image networks and the semantic components, each image branch identifies the top k positive samples most relevant to the semantics under semantic constraints. These samples are then treated as positive pairs for mutual alignment. The network is composed of three main parts: the language branch, Image Branch 1, and Image Branch 2, with Image Branch 1 and Image Branch 2 having the same network structure. The overall structure of the network is illustrated in Figure 1.

The language branch primarily consists of two components: prompt embedding and feature extraction. The prompt embedding component introduces a set of learnable vectors at the input end of the language branch to model the contextual prompts. The image branch mainly comprises four components: data augmentation, prompt embedding, feature extraction, and image patch selection. The data augmentation part performs the basic image enhancements, including random cropping, horizontal flipping, and color perturbation. This process provides similar samples to the dual-image networks, facilitating the subsequent identification of image patches with the same semantics under semantic constraints.

(1) $$X_{img}^{0}=\left[ICLS,P,I_{1},\ldots ,I_{n}\right]+E_{pos}$$

(2) $$X_{img}^{\left(l\right)}=EncoderLayer\left(X_{img}^{\left(l-1\right)},P\right)$$

(3) $$\begin{array}{c} X_{text}=\left[ICLS,P,T_{1},\ldots ,T_{n}\right]+E_{pos} \end{array}$$

In the image branch, suppose the input image, after being divided into patches, has vectors represented as $I=[I_{1},\ldots ,I_{n}]$, where n is the number of image patches. We introduce a learnable image classification token vector [ICLS] and a prompt vector [P]. Then, these are spliced together and input together into the first time of the frozen encoder, that is, Equation (1). Meanwhile, we also introduce learnable prompts in the subsequent layers of the visual encoder. The feature outputs of these layers are as described in Equation (2). In the language branch, as described in Equation (3), we define M learnable prompts and input the M learnable prompts and the TCLS generated by the text together into the text encoder for feature extraction. For feature extraction, we use the ViT-b/32 backbone network, but after the final layer of the encoder, it outputs both [ICLS] and image patch features. The output image patch features are used to calculate similarity with the output from the language branch to obtain relaxed positive samples at the image patch level. The image patches most similar to the language features are selected as positive samples with semantic information. The most semantically similar positive samples from the two branches are then brought closer together to complete the three sets of relaxation strategies under the multimodal setting.

#### 2.1.1. Relaxation of Identity Input in Multimodal Settings

The relaxation of identity input in the bimodal context consists of two parts: the prompt in the language branch and the prompt in the image branch. The language branch is composed of learnable vectors and [TLS], while the image branch, after passing through the embedding layer, consists of learnable vectors, global image features [ICLS], and image patch features. Together, the dual prompts from the language and image branches form the relaxed identity input in the multimodal context.

**Figure 2:**
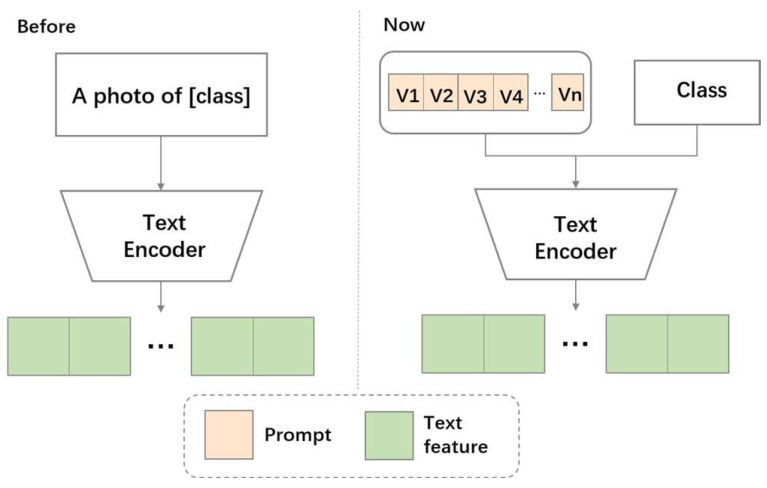
Language Prompt Diagram: the left side represents the previous prompt method, and the right side represents our prompt method.

In vision–language models (VLMs), the original image description sentence in the language branch is typically based on a handcrafted template, such as “a photo of [TCLS]”. However, due to the complexity of remote sensing images, which are difficult to describe in a single sentence, the above template definition method cannot adequately describe the content of remote sensing images, and this method adheres to a strict identity principle. Therefore, we attempt to introduce modality relaxation from the language branch. The relaxation strategy for the language branch could involve selecting the optimal text from multiple texts, fusing multiple text features, or using texts that may lack clear meaning in the real space but hold significance in the latent space. We focus on mapping the image description text to the latent space, thereby identifying the most suitable image description text in the latent space. We believe that this approach is more suitable for the subsequent task of selecting image patches under semantic guidance. Assume that N learnable vectors are defined in the language branch, represented as [V1], [V2], …, [VN]. These vectors, along with the class-labeled [TCLS], are input into the text encoder to obtain text representations, as shown in Figure 2.

**Figure 3:**
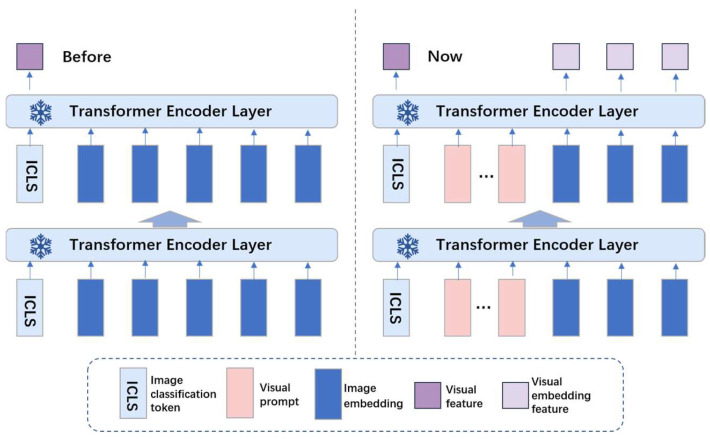
Visual Prompt Diagram: the left side represents the previous prompt method, and the right side represents our prompt method.

In the original VLM, the image branch consists of a single network. However, considering the extremely rich additional information inherent in remote sensing images, this paper argues that the information in the images should be fully explored. Therefore, a dual-network image branch is used to extract image features, with the structure of the image branch shown in Figure 3. It should provide a concise and precise description of the experimental results and their interpretation, as well as the experimental conclusions that can be drawn.

To achieve relaxation in the image branch, and inspired by the language branch, we also introduce prompts. Specifically, the backbone network selected for the image branch is ViT-b/32, which primarily consists of three components: the embedding layer, the encoder, and the classification module (MLP head). Assuming the original image size is H × W × C, the image is first divided into N image patches before entering the embedding layer. These N image patches are flattened and adjusted to two dimensions and then mapped to a d-dimensional space, where class information [ICLS] and positional information are embedded, along with the addition of P d-dimensional image prompts. After entering the encoder, P d-dimensional image prompts are added to each input space, with the encoder kept frozen during this process. After passing through the encoder, the original network extracts [ICLS] and feeds it into the classification module to classify the image categories. Simultaneously, the image patch information is output, laying the groundwork for the subsequent task of relaxed identity positive sample selection under semantic guidance.

#### 2.1.2. Semantic-Guided Relaxed Positive Sample Selection

**Figure 4:**
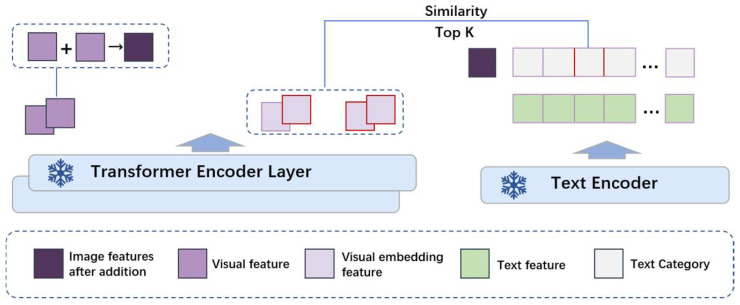
Relaxed identity positive sample selection under semantic guidance, where the red parts represent the top k image patches most similar to the semantics.

To fully utilize the information gain provided by different modalities and enhance the network’s attentiveness to additional information in remote sensing images, we propose a semantic-guided relaxed identity positive sample selection strategy, and the semantic guidance is progressively established and optimized during tuning. In the fine-tuning of CLIP, we input all textual information on the text side, as shown in Figure 1. The model then autonomously selects the text that closely aligns with the overall input image (represented by the CLIP’s image class token). This selected text further guides the choice of image patches. Our method employs a dual-network image branch. After augmenting the original images, we calculate the similarity between the text features and the image patch features extracted by each branch. The top k most similar image patches to the text are selected, and the positive samples from both network branches are treated as positive sample pairs, which are then brought closer together. This process achieves semantically guided relaxed identity positive sample selection. The network structure is illustrated in Figure 4.

The same operation is performed on both image networks. Suppose the output of the last image encoder layer Li is [*B*, *T*, *Ei*], where B represents the batch size, T represents [ICLS] and the image patches, and Ei represents the image feature dimensions. The similarity between the image patches in T and the text features [*B*, *Et*] (obtained from the text encoder) is calculated, and the top k (k ∈ [1,4]) most similar image patches to the text are selected. The reason why k is set to 4 here is that we experimented with different numbers of positive samples (1, 2, 4, and 8 positive samples) and found that the number of positive samples did not significantly impact the experimental results. Therefore, considering both computational efficiency and relative performance, we chose to set top k to 4.

The image patches extracted by Image Branch 1 are denoted as $p_{i}^{l}$ (i ∈ k), and those extracted by Image Branch 2 are denoted as $p_{i}^{2}$ (i ∈ k). The overall image features from the two branches are brought closer together, as described by Equation (4). Additionally, the top k image patches extracted by the two branches are treated as positive sample pairs and brought closer together, with the loss function given in Equation (5).

(4) $$L_{A}=-log\frac{\exp\left(\frac{sim\left(z_{i},z_{j}^{+}\right)}{\tau }\right)}{\exp\left(\frac{sim\left(z_{i},z_{j}^{+}\right)}{\tau }\right)+\sum_{j=1}^{2\left(N-1\right)}\exp\left(\frac{simsim\left(z_{i},z_{j}^{-}\right)}{\tau }\right)}$$

(5) $$L_{p}=\sum_{i=1}^{k}{\left|p_{i}^{1}-p_{i}^{2}\right|}^{2}$$

Here, $z_{i}$ is the global feature extracted by Image Branch 1, and $z_{j}^{+}$ and $z_{j}^{-}$ are the global features extracted by Image Branch 2. Equation (4) is a common loss function in contrastive learning. The goal of this loss function is to maximize the similarity of positive samples obtained by two branch networks at the feature level and minimize the similarity of negative samples simultaneously, enabling the model to better distinguish between similar and dissimilar samples. The goal of Equation (5) is to minimize the difference between the image patches obtained by Image Branch 1 and Image Branch 2. It pulls positive samples closer from the overall image perspective.

## 3. Experiments

To verify the effectiveness of our method, we have conducted a variety of experiments on four different datasets. The following are the detailed settings and results of the experiments.

### 3.1. Datasets for Test

**Figure 5:**
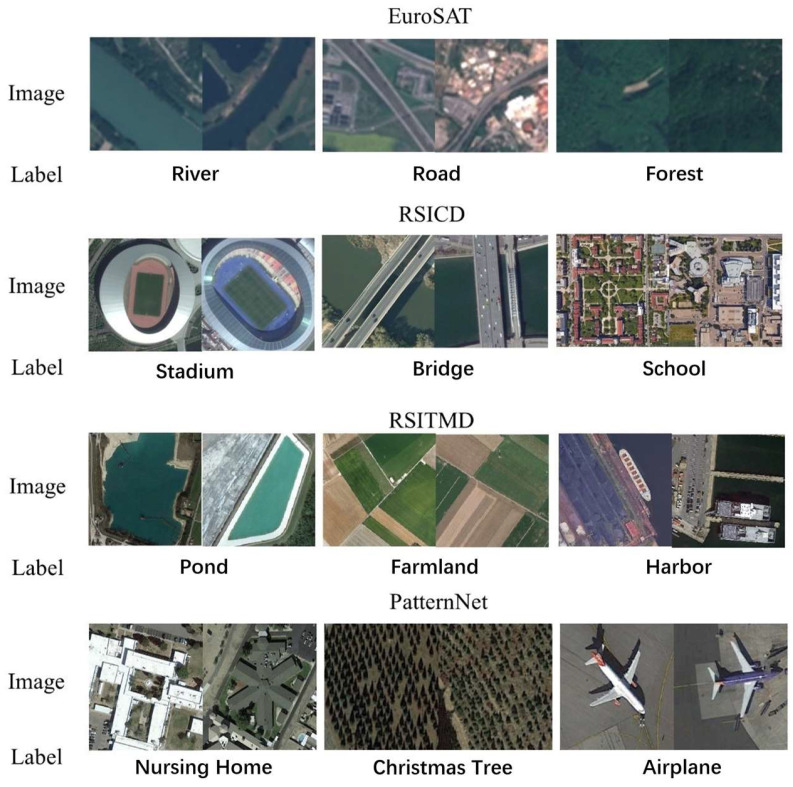
Dataset visualization results.

To better demonstrate the effectiveness of the MRiSSNet method, experiments were conducted using four datasets, each with a varying number of categories. The dataset with the fewest categories contains 10 types of land objects, while the dataset with the most categories contains 38 types. Some of the datasets include uncommon land object categories, such as resorts, Christmas tree farms, solar panels, etc. Visualizations of dataset are shown in Figure 5.

Below is an introduction to these different datasets:

1. EuroSAT [26]. The EuroSAT dataset is a land cover classification dataset containing 10 types of land objects, including industrial buildings, residential buildings, annual crops, permanent crops, rivers, seas, and lakes, herbaceous vegetation, roads, pastures, and forests. The dataset contains a total of 27,000 images, with each image sized at 64 × 64 pixels. Each scene category includes between 2000 and 3000 remote sensing images.
2. RSICD [27]. The RSICD dataset is a remote sensing image classification dataset collected from open platforms. It contains 30 types of land objects, including airports, bare land, baseball fields, beaches, bridges, central areas, churches, commercial areas, dense residential areas, deserts, cropland, forests, industrial areas, grasslands, medium-sized residential areas, mountains, parks, playgrounds, ponds, ports, railway stations, resorts, rivers, schools, sparse residential areas, squares, stadiums, storage tanks, and viaducts. The dataset contains a total of 10,921 images, with each image sized at 224 × 224 pixels.
3. RSITMD [28]. The RSITMD dataset is a remote sensing image classification dataset collected from platforms such as Google Maps and Baidu Maps. It has various resolutions and includes 30 types of land objects, such as airports, open spaces, baseball fields, bridges, churches, commercial lands, dense residential areas, deserts, cropland, forests, industrial lands, grasslands, medium-sized residential areas, mountains, parks, schools, squares, parking lots, playgrounds, ponds, viaducts, ports, railway stations, resorts, rivers, sparse residential areas, storage tanks, and stadiums. The dataset contains a total of 10,921 images, with each image sized at 224 × 224 pixels.
4. PatternNet [29]. The PatternNet dataset is a remote sensing image dataset focused on land-use types, containing 38 types of land objects, including airports, baseball fields, basketball courts, beaches, bridges, cemeteries, shrubs, Christmas tree farms, closed roads, coastal buildings, crosswalks, dense residential areas, ferry docks, football fields, forests, highways, golf courses, harbors, intersections, parks, nursing homes, oil fields, oil wells, overpasses, parking lots, parking spaces, railways, rivers, runways, runway markings, shipyards, solar panels, sparse residential areas, storage tanks, swimming pools, tennis courts, transformer stations, and wastewater treatment plants. The dataset contains a total of 30,400 images, with each image sized at 256 × 256 pixels, and each category includes 800 images.

### 3.2. Baslines

We used CoOp [25], CoCoOp [30], VPT [31], DPT [32], APPLeNet [33], C-SAW [34], and MRiSSNet-Base as baseline experiments. CoOp is a method that optimizes vision–language models through trainable textual prompts, aiming to enhance the model’s performance on specific tasks, with particularly notable improvements in few-shot learning. CoCoOp is an extended version of this approach. By introducing a conditional generation network, it dynamically adjusts the textual prompts based on the input image. DPT is a multimodal contrastive learning network that uses dual-modal prompt tuning, incorporating both textual and visual prompts into its architecture. Additionally, it introduces a class-aware visual prompt adjustment strategy. By feeding image and text information into the CAVPT generator, DPT encodes task-related information and applies a cross-attention mechanism to generate dynamic class-aware visual prompts. This enables the network to implement a relaxation strategy in a multimodal context. APPLeNet uses CLIP-base prompt learning with multi-scale visual features and style info; anti-correlation regularization enhances cross-domain generalization. C-SAW is an improvement based on APPLeNet, and C-SAW uses self-supervised prompt learning with visual attention and jigsaw reconstruction; multi-loss optimization boosts generalization across domains

MRiSSNet-Base is a fundamental relaxed identity contrastive learning framework for remote sensing image feature extraction under the relaxed identity assumption. This model is a simplified version of our proposed method, incorporating learnable visual cues and language cues into the network.

### 3.3. Basic Experimental Setup

We compare the results of our method (MRiSSNet) with those of various baseline experiments. All experiments were conducted with identical parameter settings: the 1-shot training cycle was 60 epochs, while the 2/4/8/16-shot training cycles each consisted of 100 epochs. The batch size was set to 128, and the optimizer used was SGD. For methods with language prompts, the prompt length was uniformly set to 16, as in CoOp, CoCoOp, DPT, and our methods (MRiSSNet-Base and MRiSSNet). For methods with image prompts, the prompt length was uniformly set to 10, such as in VPT, DPT, and our methods (MRiSSNet-Base and MRiSSNet).

### 3.4. Experimental Results and Analysis

#### 3.4.1. Classification Experiment

(1) One-shot

We compared the proposed method with the results of various baseline experiments. To highlight the advantages of the multimodal model, this section presents the one-shot experimental results, as shown in Table 1.

As shown in Table 1, MRiSSNet-Base had lower metrics across all four datasets compared to MRiSSNet. However, when compared to other baselines, MRiSSNet-Base outperformed the best baseline methods in certain metrics on the RSITMD and PatternNet datasets. Specifically, on the RSITMD dataset, MRiSSNet-Base surpassed the best baseline methods (DPT, CoOp) by 0.9% in accuracy (Acc), although it Macro-F1 score was 1% lower. On the PatternNet dataset, MRiSSNet-Base exceeded the best baseline method (DPT) by 3.2% in Acc and 3.5% in Macro-F1. After incorporating the tripartite relaxed positive sample strategy, the network’s performance across all datasets surpasses that of MRiSSNet (Base), further demonstrating the effectiveness of the strategy proposed in this paper. This is within expectations because, unlike natural images, for remote sensing images, light, texture, etc., in the image are very important features. This is also one of the issues considered in the design of our method. Our method is expected to more fully express the information contained in remote sensing images, just as we stated in the introduction. However, methods such as VPT and CoOp, which are designed based on natural images, have neglected the attention to this information. Similarly, APPLNet and S-CAW also introduced prompts. However, they mainly focus on the extraction of multi-scale features of images or the contextual image information, without paying attention to the constraints existing between image–text pairs. Therefore, their performance on PatterNet and RSICD is inferior to ours.

Among the baseline methods, excluding MRiSSNet-Base, the DPT method demonstrates relative stability, indicating that dual-modal prompting is an effective prompting method. Moreover, our method achieved better results across all four datasets. On the EuroSAT dataset, our method had an Acc that was 7.8% lower. On the RSICD dataset, our method outperformed the best baseline method by 2.6% in Acc and 2.3% in Macro-F1. On the RSITMD dataset, our method was 0.4% higher in Acc but 0.7% lower in Macro-F1 compared to the best baseline. On the PatternNet dataset, our method outperformed the best baseline by 3.7% in Acc and 4.1% in Macro-F1. Our method outperformed all baseline experiments in all metrics on two of the datasets and outperformed the best baseline methods in certain metrics on the other two datasets. The most significant improvement was observed on the PatternNet dataset, where our method achieved an Acc of 91.1% with just one shot. Analysis indicates that the PatternNet dataset is relatively balanced in terms of data distribution, and the image quality is also good.

(2) Few-shot

**Figure 6:**
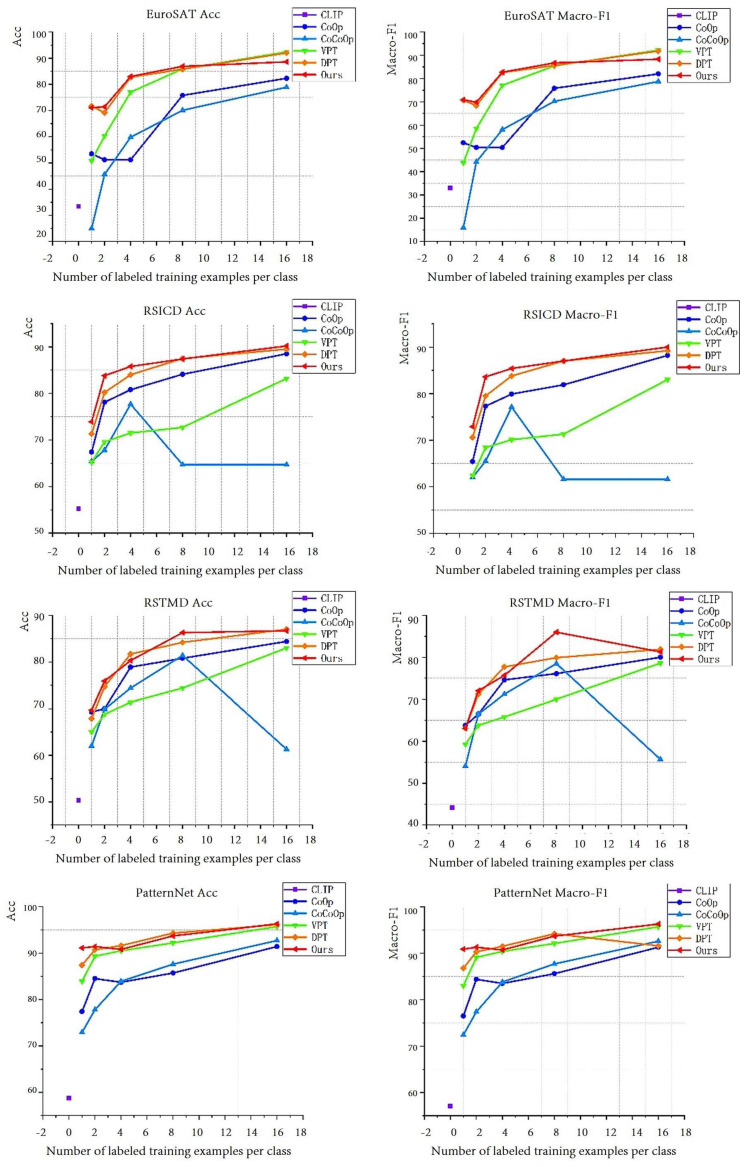
Experimental results of various methods on different datasets for 2/4/8/16 shots.

In addition to the 1-shot experiment, we conducted 2/4/8/16-shot experiments to explore whether our method maintains strong discriminative ability under few-shot settings. The results, shown in Figure 6, depict different methods using different colored lines, with our method represented by the red line. As seen in Figure 6, our method achieved better results across all datasets, regardless of whether the metric was Acc or Macro-F1. It is worth noting that the one-shot results of all methods significantly outperform the zero-shot results of the CLIP method, which indicates that the CLIP model may not be suitable for remote sensing datasets. Additionally, the one-shot results of the language-prompting method CoCoOp were consistently the worst across all datasets. In two datasets, there was a trend of decreasing accuracy as the number of prompt samples increased.

On the EuroSAT dataset, all methods except CoOp showed an upward trend in metrics as the number of prompt samples increased. Our method consistently outperformed the DPT method up to the 16-shot experiment, where DPT surpassed our method. Interestingly, the CoOp method showed a decreasing trend in accuracy from one shot to four shots as the number of prompt samples increased. On the RSICD dataset, our method outperformed all other methods, making it the best learning approach. Notably, from one shot to two shots, our method showed an improvement of nearly 10% in both Acc and Macro-F1. However, CoCoOp experienced a significant downturn during the eight-shot experiment, with a sharp decline in metrics after an initial increase. On the RSITMD dataset, our method performed particularly well in the two-shot and eight-shot settings. In the eight-shot experiment, it outperformed the second-best method by 6.1% in Macro-F1. It is noteworthy that CoCoOp experienced another sharp decline in the 16-shot experiment on this dataset, resulting in performance almost identical to its one-shot results. On the PatternNet dataset, the performance of various methods was relatively stable, showing a gradual upward trend. Our method was the best in the 1-shot and 16-shot settings. However, it slightly lagged behind the DPT method in the two-shot and eight-shot experiments.

In summary, across the 2/4/8/16-shot methods, approaches that rely solely on visual prompts or language prompts exhibited instability. This was particularly evident in the CoCoOp method, which uses language prompts and showed a dramatic decline in performance as the number of prompt samples increased. In contrast, dual-prompt methods like DPT and MRiSSNet demonstrated a more stable growth trend, with the rate of improvement transitioning from rapid to gradual. This also highlights the stability of our proposed method.

#### 3.4.2. Experiment on Relaxation of Identity Input in a Multimodal Setting

To validate the necessity of the bimodal relaxation strategy, this section retains the semantically guided relaxed identity sample strategy while individually removing the language relaxation input and the visual relaxation input from the network. This allows us to evaluate the performance gains when only one type of relaxation input method is used. The experimental results are shown in Table 2, where “No_L” represents the absence of the language relaxation input strategy, with only the visual relaxation input strategy applied; “No_V” represents the absence of the visual relaxation input strategy, with only the language relaxation input strategy applied; and “Ours” represents our method. Experiments were conducted on four datasets, with the length of the language prompt set to 16 and the length of the visual prompt set to 10 in all experiments.

In this section, we take the one-shot experimental results as an example. As shown in Table 2, the best performance is achieved on three datasets when both language prompts and image prompts are used. However, in the RSITMD dataset, performance improves when the language prompt is removed. This may be related to the relatively small amount of data per class in the RSITMD dataset, where each class contains only around 100 images. Additionally, this section observes a significant drop in performance when the visual prompt is removed, which aligns with our hypothesis. We believe that the visual aspect of remote sensing images contains more abundant information, which cannot be fully replaced by language.

To further explore the effects of language prompts and image prompts, experiments were conducted using different lengths of language prompts and visual prompts, all within the one-shot setting. The experimental results are shown in Table 3 and Table 4.

As with the results of other works, different tasks will have different optimal prompt lengths. Meanwhile, we notice that in both visual and textual prompts, prompt length has a more significant effect on the EuroSAT. We hypothesize that this is due to differences in the number of images per category within EuroSAT, which may lead to instability in model learning, particularly for categories with relatively small amounts of data. Additionally, the lower resolution and smaller size of EuroSAT images make it challenging for models like CLIP to capture sufficient detail, hindering the learning of strong visual features. Fine-tuning CLIP to adapt to these data is a process of adjusting learnable prompts. Therefore, these instabilities make the model particularly sensitive to prompt length during fine-tuning. In contrast, datasets such as RSICD, RSITMD, and PatterNet may have a more balanced category distribution and larger image sizes, making the model less sensitive to variations in prompt length. At the same time, across different datasets, the model is more sensitive to changes in the length of textual prompts than visual prompts. We believe this is because, compared to images, textual information is more abstract, and changes in prompt length mean the addition or loss of more semantic information.

#### 3.4.3. Experiment on Relaxation of Semantic-Guided Positive Sample Selection

To validate the necessity of the semantically guided relaxed identity sample strategy, this section retains the language and visual relaxation inputs but removes the semantically guided relaxed identity module. Experiments were conducted on four datasets, and the results are shown in Table 5. “No_VandL” indicates the removal of the semantically guided relaxed identity sample strategy. The table presents the experimental results for the one-shot setting, with the visual prompt length set to 16 and the language prompt length set to 10. As shown in Table 5, across the four datasets, our method consistently achieves the best experimental results compared to the model without the semantic guidance module. On average, there is an improvement of approximately 3% in Acc and 4.7% in Macro-F1. The experiment with semantically guided relaxed identity samples showed the most significant improvement in the RSICD dataset, with a 4.4% increase in Acc and a 3.6% increase in Macro-F1. The smallest improvement was observed in the RSITMD dataset, with only a 2.3% increase in Acc and a 0.6% increase in Macro-F1.

**Figure 7:**
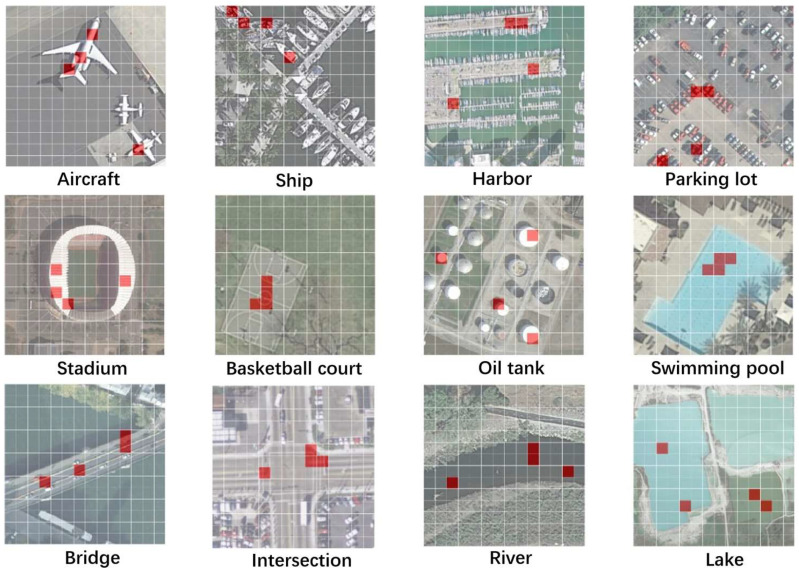
Visualization of results for relaxed identity sample selection under semantic guidance.

To further examine the accuracy of positive sample selection under semantic constraints, this section visualizes some of the selected positive sample regions, as shown in Figure 7. As shown in Figure 7, the positive samples selected based on semantic guidance are quite accurate, with more clearly defined semantic information compared to those selected through unimodal self-supervised contrastive learning. Additionally, we observed that for the images of airplanes and oil tanks, the network selected airplanes and oil tanks of different scales, and for lakes, it selected lakes of different colors displaying significant variation. These positive samples could not have been obtained through traditional data augmentation methods. Furthermore, this approach overcomes the lack of semantic constraints in positive samples typical of unimodal contrastive learning, enabling relaxed positive sample selection guided by semantics rather than relying on prior knowledge or network-learned feature constraints.

## 4. Conclusions

Based on the relaxed identity assumption, we summarize the threefold relaxation constraints in multimodal contrastive learning: relaxation of positive samples for visual input, language input, and the fusion of visual and language inputs. We propose a multimodal prompt-tuning strategy combined with a semantic-guided positive sample selection method. Our approach surpasses the current state-of-the-art methods in visual, language, and visual–language interaction domains, such as CLIP, CoOp, CoCoOp, VPT, and DPT. Additionally, we validated the effectiveness of our method on four remote sensing datasets and visualized the selected regions of positive samples guided by semantics.

## 5. Discussion

Due to the complexity and uncertainty of remote sensing images, we use prompt-tuning and relaxed positive sample selection with semantic guidance to help the model learn strong multimodal feature representations. Through this method, we find that multimodal prompts are superior to unimodal ones, which indicates that when conducting contrastive learning in a multimodal setting, we need to combine the two modalities to give full play to the advantages of the multimodal model, thereby achieving better feature extraction. Moreover, there are still some aspects of our method that can be improved. For example, the selection of our semantically guided positive samples is at the patch level, which means that negative samples may still be included in the sampled positive samples. Is it possible to be more precise to a finer-grained level to obtain more accurate positive and negative samples? In addition, the semantic guidance here is at the object level, and important image information, such as object attributes like color, contour, and pattern, is not included in the scope of semantic constraints. Finally, we construct positive samples through a two-branch network, which implies a greater computational cost. How to construct a more efficient network structure is also an aspect worthy of exploration.

## Figures and Tables

**Table 1:** Classification results of various multimodal contrastive learning methods on four datasets (CLIP for zero-shot and the other methods for one-shot).

Method	EuroSAT		PatternNet		RSICD		RSITMD	
	Acc	Macro-F1	Acc	Mcro-F1	Acc	Macro-F1	Acc	Mcro-F1
CLIP-Base	36.1	32.6	53.5	49.8	54.9	48.0	48.1	42.9
CLIP-Prompt	33.4	33.1	58.8	57.1	55.2	48.6	50.3	44.2
CoOp	53.5	52.4	77.4	76.5	67.4	65.4	69.3	**63.8**
CoCoOp	25.0	15.9	72.9	72.4	65.4	62.0	62.0	54.1
VPT	50.8	43.8	83.9	83.0	65.2	62.4	65.0	59.3
DPT	71.7	70.7	87.4	86.8	71.3	70.6	67.8	63.1
APPLeNet	75.46	-	65.57	-	60.71	-	-	-
C-SAW	**77.20**	-	70.18	-	62.56	-	-	-
MRiSSNet-Base	69.4	70.2	90.6	90.3	71.1	69.4	68.7	62.8
MRiSSNet	71.1	**70.9**	**91.1**	**90.9**	**73.9**	**72.9**	**69.7**	63.1

The highest scoring metrics are highlighted in bold.

**Table 2:** Results of the experiment on relaxed identity in a multimodal setting.

Method	EuroSAT		RSICD		RSITMD		PatternNet	
	Acc	Macro-F1	Acc	Macro-F1	Acc	Mcro-F1	Acc	Mcro-F1
No_L	68.1	67.5	70.7	69.3	**71.2**	**67.0**	75.6	73.0
No_V	32.5	26.8	67.9	64.9	66.2	61.8	86.5	86.1
Ours	**71.1**	**70.9**	**73.9**	**72.9**	69.7	63.1	**91.1**	**90.9**

The highest scoring metrics are highlighted in bold.

**Table 3:** Results of the experiment on changing the length of visual prompts.

Prompt Length	EuroSAT		RSICD		RSITMD		PatternNet	
	Acc	Macro-F1	Acc	Macro-F1	Acc	Mcro-F1	Acc	Mcro-F1
5	61.1	60.6	71.2	69.1	64.9	60.5	89.3	88.8
10	**71.1**	**70.9**	**73.9**	**72.9**	69.7	63.1	**91.1**	**90.9**
15	40.5	35.9	72.6	69.9	**69.8**	**64.0**	89.6	89.3
20	45.1	41.2	71.0	70.0	71.5	67.0	89.4	88.8

The highest scoring metrics are highlighted in bold.

**Table 4:** Results of the experiment on changing the length of language prompts.

Prompt Length	EuroSAT		RSICD		RSITMD		PatternNet	
	Acc	Macro-F1	Acc	Macro-F1	Acc	Mcro-F1	Acc	Mcro-F1
4	59.9	58.1	66.9	65.3	**70.7**	**66.6**	88.2	87.9
8	48.3	43.2	68.5	65.8	69.2	62.0	87.7	87.2
16	71.1	70.9	**73.9**	**72.9**	69.7	63.1	**91.1**	**90.9**
32	**82.3**	**82.0**	71.9	71.3	70.1	65.3	86.6	84.7

The highest scoring metrics are highlighted in bold.

**Table 5:** Results of the experiment on relaxed identity samples under semantic guidance.

Training Method	EuroSAT		RSICD		RSITMD		PatternNet	
	Acc	Macro-F1	Acc	Macro-F1	Acc	Mcro-F1	Acc	Mcro-F1
No_VandL	68.1	66.6	69.5	69.3	67.4	62.5	88.7	88.3
Ours	**71.1**	**70.9**	**73.9**	**72.9**	**69.7**	**63.1**	**91.1**	**90.9**

The highest scoring metrics are highlighted in bold.

## Data Availability

Data are contained within the article.
